# Structure-Property Relationship on the Example of Gas Separation Characteristics of Poly(Arylene Ether Ketone)s and Poly(Diphenylene Phtalide)

**DOI:** 10.3390/membranes11090677

**Published:** 2021-08-31

**Authors:** Alexandre Alentiev, Sergey Chirkov, Roman Nikiforov, Mikhail Buzin, Oleg Miloserdov, Victoria Ryzhikh, Nikolay Belov, Vera Shaposhnikova, Sergey Salazkin

**Affiliations:** 1 A.V. Topchiev Institute of Petrochemical Synthesis, Russian Academy of Sciences (TIPS RAS), 119991 Moscow, Russia; sm1th@ips.ac.ru (S.C.); nru@ips.ac.ru (R.N.); kochenkova@ips.ac.ru (V.R.); belov@ips.ac.ru (N.B.); 2 A.N. Nesmeyanov Institute of Organoelement Compounds, Russian Academy of Sciences (INEOS RAS), 119334 Moscow, Russia; buzin@ineos.ac.ru (M.B.); vsh@ineos.ac.ru (V.S.); snsal@ineos.ac.ru (S.S.); 3 V.A. Trapeznikov Institute of Control Sciences, Russian Academy of Sciences (ICS RAS), 117997 Moscow, Russia; oleg_milos@mail.ru

**Keywords:** polyether ketones, polymer synthesis, gas separation membranes, permeability, diffusion, properties prediction

## Abstract

Three poly(arylene ether ketone)s (PAEKs) with propylidene (C1, C2) and phtalide (C3) fragments, and one phtalide-containing polyarylene (C4), were synthesized. Their chemical structures were confirmed via ^1^H NMR, ^13^C NMR and ^19^F NMR spectroscopy. The polymers have shown a high glass transition temperature (>155 °C), excellent film-forming properties, and a high free volume for this polymer type. The influence of various functional groups in the structure of PAEKs was evaluated. Expectedly, due to higher free volume the introduction of hexafluoropropylidene group to PAEK resulted in higher increase of gas permeability in comparison with propylidene group. The substitution of the fluorine-containing group on a rigid phtalide moiety (C3) significantly increases glass transition temperature of the polymer while gas permeation slightly decreases. Finally, the removal of two ether groups from PAEK structure (C4) leads to a rigid polymer chain that is characterized by highest free volume, gas permeability and diffusion coefficients among the PAEKs under investigation. Methods of modified atomic (MAC) and bond (BC) contributions were applied to estimate gas permeation and diffusion. Both techniques showed reasonable predicted parameters for three polymers while a significant underestimation of gas transport parameters was observed for C4. Gas solubility coefficients for PAEKs were forecasted by “Short polymer chain surface based pre-diction” (SPCSBP) method. Results for all three prediction methods were compared with the ex-perimental data obtained in this work. Predicted parameters were in good agreement with ex-perimental data for phtalide-containing polymers (C3 and C4) while for propylidene-containing poly(arylene ether ketone)s they were overestimated due to a possible influence of propylidene fragment on indices of oligomeric chains. MAC and BC methods demonstrated better prediction power than SPCSBP method.

## 1. Introduction

Over the past twenty years, the separation of gas mixtures has been one of the most rapidly developing and knowledge-intensive areas of membrane technology. The following different types of materials are used to prepare membranes for gas separation: polymeric materials, such as homo- and copolymers [1]; inorganic materials, such as carbon molecular sieves [2] or metals [3]; and polymer matrices filled with inorganic particles, such as so-called mixed-matrix membranes [4]. In the majority of membrane gas separation processes, asymmetric or composite polymer membranes are used, the materials of the thin selective layer [5] of which, as a rule, are glassy amorphous polymers.

Mass transfer in such membranes is carried out by the solution-diffusion mechanism. The stationary gas flow through the membrane is directly proportional to the pressure drop and inversely proportional to the thickness of the membrane. In this case, the proportionality coefficient is the gas permeability coefficient (P) for the material of the selective membrane layer. In the literature, P is usually expressed in off-system units, i.e., Barrer (1 Barrer = 10^−10^ cm^3^(STP) cm cm^−2^ s^−1^ cmHg^−1^). The P values for polymer materials vary within 3–8 orders of magnitude, depending on the nature of the polymer and the gas [5,6]. P for the polymer–gas system is determined by the gas diffusion coefficient D and gas solubility coefficient S of the material [6], as follows:

P = D·S,(1)

In the literature on the membrane topic, as a rule, D is expressed in cm^2^ s^−1^, and S is expressed in cm^3^(STP) cm^−3^·cmHg^−1^ or in cm^3^(STP) cm^−3^·atm^−1^.

An important factor characterizing the efficiency of the gas separation process is the ideal selectivity of the separation of i and j gases, as follows:

α_ij_ = P_i_/P_j_, (2)

The potential efficiency of the material for gas separation is determined by the position of the experimentally measured values P and α for the polymer on the permeability–selectivity diagrams (Robeson diagrams), relative to the empirical so-called “upper bounds” [7,8] of the overall distribution. The “upper bounds” on the Robeson diagrams are formed by polymers with the most-favorable combination of permeability and selectivity. The coefficients of permeability, diffusion, and solubility, as well as the ideal selectivity of gas separation, are considered to be the transport parameters of the polymer–gas system, at a constant temperature. To separate mixtures of non-condensable gases, such as H_2_, He, N_2_, O_2_, CO_2_, and CH_4_, glassy amorphous polymers with high diffusion selectivity are used as materials for the selective layer [6,9].

The chemical structure of a polymer affects its macroscopic properties in a complex way. Firstly, it is connected to the intermolecular interactions in the polymer and its chain rigidity. These properties subsequently determine the glass transition temperature, density, free volume, and other macroscopic characteristics of the polymer [6,10]. Moreover, the latter, in turn, have an influence on the gas transport properties. Thus, most of the works on the design of the chemical structure of polymers are based on the assumption that a higher fractional free volume (and lower density) leads to higher gas permeability, while free volume distribution and also intermolecular interactions have an effect on the selectivity of different gas pairs [6]. For a long time, the main direction to improve gas transport characteristics consisted of an introduction of bulky moieties into a polymer structure [11]. However, the latest trends in high-performance polymer preparations include the structures with “kink” fragments, e.g., PIMs, polymers with a bisfluorene fragment, Tröger base polymers, and iptycene-containing polymers [12,13,14]. Thus, phtalide-containing polymers seem relevant for the study of such structures.

The extensive information accumulated to date on the transport parameters of polymers with different chemical structures of the elementary unit [15,16], allows one to not only search for empirical relationships between the chemical structure of the elementary unit with the transport characteristics of polymers [7,8,13,17,18,19,20,21,22,23,24,25,26,27,28], but also to predict transport parameters from the chemical structure of the elementary unit [29]. Such predictions are possible both by the group contribution methods [29,30,31,32,33,34,35,36,37,38,39], but also by using elements of the graph theory [29,40,41,42] and geometrical indices [43,44], as well as by using neural networks [29,45,46] or computer modeling [29,47,48,49,50,51,52,53,54,55,56]. Computer modeling by molecular dynamics, or Monte Carlo [50,55,56], is a fast-developing method for predicting the transport parameters of polymers, and, from the point of view of the physical nature of the process, the most adequate one. However, these methods take a lot of time for calculations on supercomputers and, with all the prospects, they cannot currently be used for a wide range of polymers, although they seem necessary for modeling local tasks for specific polymer materials. The use of self-learning neural networks to predict the properties of substances requires large datasets (several tens of thousands of structures studied), which is unreachable for the transport parameters of polymers; currently, no more than 2000 structures have been studied. Therefore, the use of neural networks for the adequate prediction of the transport parameters of polymers is currently limited. More attractive for the existing datasets [15,16] are the various variants of the group contribution method that was used for these datasets before [29,35,36,38], or the developed methods of geometrical indices [43,44], which takes into account the geometric parameters of the model fragments of polymer chains. However, when using any prediction methods, it is necessary to verify these methods for new polymers that were not included in the dataset, on the basis of which these contributions or indices were determined.

Among the classes of polymers presented in the database [15], not all are equally present. Polyimides have the most numerous representation (Table 1); therefore, for this class, the predicted data are closest to the experimental ones [29,32,35]. For such a practically important and extensive class of polymers as poly(arylene ether ketone)s [57,58], the database [15] contains only 41 different structures, so for this class, the verification of predicted data is very interesting.

Thus, in this work, a number of poly(arylene ether ketone)s (PAEKs), as well as poly(diphenylene phthalide), which only has a common structural element with one of the PAEKs, were synthesized and studied, and the process of verifying various methods for predicting transport parameters for these polymers was carried out.

## 2. Materials and Methods

### 2.1. Polymer Synthesis

**Figure 1:**
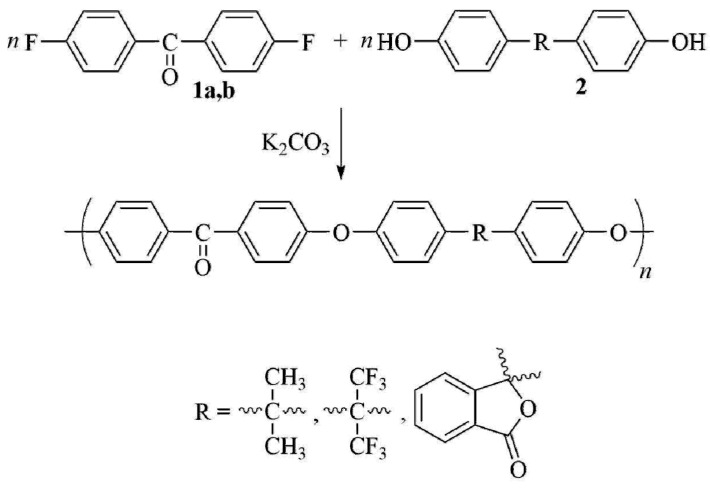
PAEKs C1–C3 synthesis scheme.

The synthesis of poly(arylene ether ketone)s (PAEKs: C1–C3, Figure 1) was carried out by polycondensation method according to the mechanism of the reaction of nucleophilic substitution of activated halogen in arylene dihalide, by the interaction of 4,4′-difluorobenzophenone with dipotassium phenolates of bisphenols (bisphenol A, bisphenol AF, phenolphthalein), similar to the method described in [59] (Figure 1). Further, 4,4′-Difluorobenzophenone (0.0988 mol), bi-sphenol(2,2-bis(4′-hydroxyphenyl)propane or 2,2-bis(4′-hydroxyphenyl)hexafluoropropane or 3,3-bis(4′-hydroxyphenyl) phthalide) (0.1 mol), 4-fluorobenzophenone (0.0024 mol), pre-milled freshly calcined K2CO3 (0.13 mol), N,N-dimethylacetamide (200 mL), and chlorobenzene (100 mL) were loaded in an argon-blown four-necked flask equipped with a stirrer, an argon supply tube, and a system for azeotropic removal of water. The flask was heated on an oil bath whose temperature was increased within ~0.5 h up to 185 °C. The time of synthesis after complete removal of an azeotropic chlorobenzene–water mixture was 7 h. The reaction mixture was cooled and dissolved in chloroform. The resulting solution was filtered from salts and washed with stirring many times with water. After evaporation of the chloroform solution at 25 °C and drying for 18 h with a gradual increase in the temperature from 60 to 140 °C, and then for 25 h at 160 °C. The PAEKs was obtained as a film in yield of 99%; the reduced viscosities (ηred) were 0.62 dL/g (for C1), 0.53 dL/g (for C2) and 0.78 dL/g (for C3).

**Figure 2:**
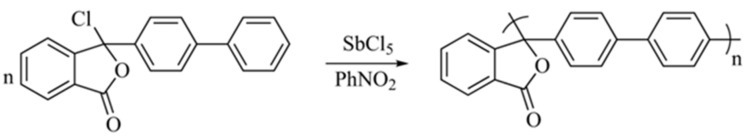
Poly(diphenylene phthalide) C4 synthesis scheme.

Reduced viscosity η_red_ was determined in chloroform at 25 °C and a polymer concentration of 0.5 g for 100 mL of a solvent, using an Ubbelohde viscometer with a capillary diameter of 0.6 mm. The synthesis of polydiphenylene phthalide was carried out by the following scheme [60] (Figure 2).

The three-necked flask (50 cm^3^) equipped with a stirrer, a gas-inlet tube and air condenser with bubble counter was purged with argon. Then 2′-carboxy-4-phenylbenzophenone acid pseudo chloride (10 g) and nitrobenzene (15 mL), which was purified by vacuum distillation and dried over molecular sieves, were placed into the flask. The solution obtained was heated in inert atmosphere to 80 °C (in bath) and then 0.4 mL of SbCl_5_ (it was synthesized by chlorination of SbCl_3_ and purified by vacuum distillation) was added. Polycondensation was carried out at 80 °C for 30 h and then at 100 °C for 10 h.

After completion of synthesis and cooling of reaction mixture, polymer was dissolved in 300 mL of purified chloroform. Polymer solution was filtered and the polymer was precipitated at stirring and slow adding in methanol (near 1000 mL of methanol purified by distillation). The precipitate obtained was filtered and washed with methanol. Then repeated extractions by methanol and acetone (methanol and acetone were purified by distillation) were performed. The polymer was dried at 120 °C. The polymer yield was 8.2 g. Polymer reduced viscosity was 0.56 dL/g at 25 °C (0.5 g of polymer per 100 mL of the chloroform).

### 2.2. Polymer NMR Characterization

Chemical structures of C1–C4 polymers were confirmed using ^1^H NMR, ^13^C NMR and ^19^F NMR spectra. Spectra for C1 and C3 were previously reported in [61], spectra for C4 were previously reported in [62].

The ^1^H and ^13^C NMR spectra of C1–C3 samples were measured on an Avance 400 Bruker spectrometer (400.13 and 100.61 MHz, respectively) using CDCl_3_ solutions; Me_4_Si was used as an internal standard. Signals in the ^1^H and ^13^C NMR spectra were assigned according to the data calculated using program ACDLabs. ^19^F NMR for C2 sample was recorded on an Avance 300 Bruker spectrometer (282.40 MHz) (Billerica, MA, USA) for solution in CDCl_3_ using CF_3_COOH as the external standard.

Results of ^1^H NMR and ^13^C NMR analyses are provided in Tables S1 and S2 (Supplementary Materials), respectively.

The ^19^F NMR spectrum of sample C2 contains a singlet in the region of minus 63.90 ppm, which is characteristic of the chemical shift of the CF_3_ group.

### 2.3. Investigation Methods

Polymer films were obtained by casting on a cellophane support from a 5% solution in chloroform (reagent grade) with drying for 2–3 days at room temperature, followed by bringing it to a constant mass in vacuum.

Differential scanning calorimetry (DSC) studies of the films obtained were performed on a DSC-3 apparatus (Mettler-Toledo, Columbus, OH, USA) equipped with a liquid nitrogen cooling system. Measurements were performed in dry argon with purging through the cell at a gas flow rate of 60 mL/min and at a heating rate of 10 °C/min. The temperature signal was calibrated by the beginning of the melting of indium and zinc at a heating rate of 10 °C/min. The heat flow rate was calibrated considering the melting heat of indium. To assess the presence of residual solvent in films, TGA method in air at a heating rate of 10 °C/min was used (Derivatograth-C apparatus, MOM Budapest, Hungary).

The density of polymer films ρ (in g/cm^3^) was determined at room temperature (22 ± 2 °C) by hydrostatic weighing in propanol-2. The fractional free volume (FFV) was determined using the Bondi method [63], as follows:

FFV = 1 − 1.3·V_w_/V_sp_, (3)

where V_w_ is the van der Waals volume of a monomer unit (cm^3^/mol); V_sp_ = M/ρ is the specific occupied volume of a polymer (cm^3^/mol); M is the molecular mass of the monomer unit of a polymer (g/mol).

The gas (He, H_2_, O_2_, N_2_, CO_2_, CH_4_) permeability (P) and diffusion (D) coefficients at a temperature of (21 ± 2 °C) for the prepared films were obtained by the integral barometric method on the MKS Barotron installation. The software based on “LabVIEW” was used to control the experiment. The experiments were carried out at room temperature; upstream pressure was about 1 atm. In the case of studying the permeability of oxygen, the upstream pressure was varied in range of 1–5 atm, and it was shown that the permeability coefficients of this gas, as well as the diffusion coefficients, remained constant. The downstream pressure did not exceed 1.3 × 10^−3^ atm; therefore, under the conditions of the experiment, the reverse diffusion of the penetrating gas was neglected. Permeability coefficient P and diffusion coefficient D were determined via the curve of gas permeation through the polymer film into a calibrated volume (P: by the slope of the linear dependence of the flow through a film after the steady-state mass transfer was reached; D: by the method of Daynes-Barrer using the time lag θ (s): D = l^2^/6θ, where l is the film thickness). The solubility coefficients (S) were calculated from the experimental values of P and D using the formula S = P/D. From the data obtained, ideal separation selectivities (α = Pi/P_j_) and diffusion selectivities (α_D_ = Di/D_j_) were found for different i and j gas pairs. The experimental measurement error for P was 5%, for D was 10% and, subsequently, the calculation errors were 15%, 10%, and 20% for S, α and α_D_, respectively.

The experimental P and D were extrapolated to 35 °C using the method from [64] in order to perform comparison with the predicted values based on the database [15].

### 2.4. Prediction Methods

#### 2.4.1. The Method of Modified Atomic Contributions (MAC) and the Method of Bond Contributions (BC)

The methods used in this work to predict gas permeability and diffusion coefficients were described previously [32,38]. Summary is provided below.

**Figure 3:**
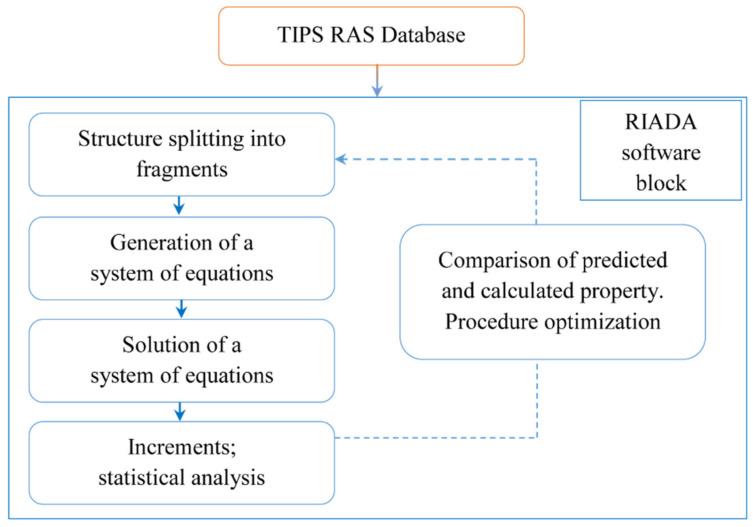
Universal flowchart for group contribution methods (MAC and BC).

A set of polymers is selected from the database [15]. All structures of repeat units are presented as molecular graphs in the database [15]. Structural formula of the repeat unit is then split into a set of fragments (atoms or bonds with the nearest neighbors), and an increment (variable, representing quantitative contribution of the fragment) of a physical property in question is assigned to every fragment. The sum of these increments multiplied by the normalization factor (introduced to consider a difference in polymer-chain molecular weights or lengths) is the predicted value of the physical property being calculated. The system of such equations (Equations (4) and (5) for MAC and BC, respectively) for a set of polymers selected is then solved to get a number of increment values and a number of predicted property values. The increments obtained are then used to calculate a new polymer property. All of the operations described above from the splitting of the polymers from the dataset to the calculation of increments and predicted properties are performed via RIADA software [38]. The difference between the modified atomic contributions (MAC) method and bond contribution (BC) method is in the way of repeat unit splitting into the fragments. In both cases the polymer repeat unit is split into the atoms (MAC) or bonds (BC) of the main chain and side groups; however, BC method also includes the “mixed” bonds connecting main chain with the side groups. The universal scheme for MAC and BC methods is provided in Figure 3.

(4) $$\left\{\begin{array}{c} lgX_{1}=\underset{i}{\sum }\left(\frac{m_{i}n_{i1}}{M_{1}}\cdot I_{i}\right)\\ lgX_{2}=\underset{i}{\sum }\left(\frac{m_{i}n_{i2}}{M_{2}}\cdot I_{i}\right)\\ \ldots \\ \ldots \\ \ldots \\ lgX_{k}=\underset{i}{\sum }\left(\frac{m_{i}n_{ik}}{M_{k}}\cdot I_{i}\right) \end{array}\right.$$

(5) $$\left\{\begin{array}{c} lgX_{1}=\underset{i}{\sum }\left(\frac{n_{i1}}{N_{1}}\cdot I_{i}\right)\\ lgX_{2}=\underset{i}{\sum }\left(\frac{n_{i2}}{N_{2}}\cdot I_{i}\right)\\ \ldots \\ \ldots \\ \ldots \\ lgX_{k}=\underset{i}{\sum }\left(\frac{n_{ik}}{N_{k}}\cdot I_{i}\right) \end{array}\right.$$

where *k* is number of polymers in test set; *i* is total number of group types (atoms for MAC or bonds for BC); *I* is an increment of a given group; *X* is a physicochemical property tested (permeability or diffusion coefficient); *n* is number of groups in a given polymer; *m* is molar mass of atom; *M* is molar mass of a monomer unit; *N* is total number of bonds in a monomer unit.

#### 2.4.2. Short Polymer Chain Surface-Based Prediction

**Figure 4:**
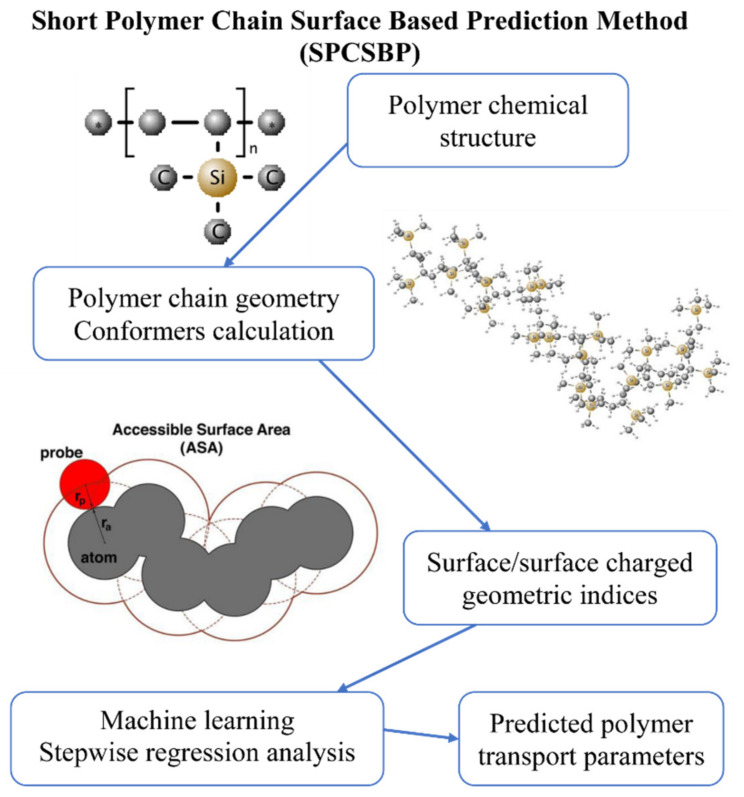
SPCSBP method flowchart.

This paper continues the development of practical application of the quantitative structure–property relationships (QSPR)-based short polymer chain surface-based prediction (SPCSBP) method from [43,44,65]. The method consists of modeling of the polymer macromolecule conformations with a size of about several hundred atoms. The approach is to calculate the accessible surface areas of the spherical model of a molecule using the Lee-Richards algorithm [66] for a large set of conformations of individual polymer chains and in constructing the dependencies of the accessible surface area (ASA) from the probe radius (Figure 4) on their basis. The coefficients of linear approximation of the obtained dependencies are used as explanatory variables in multiple linear regression. The significant variables and their weights in the regression are found based on experimental measurements from the database of the A.V. Topchiev Institute of Petrochemical Synthesis of the Russian Academy of Sciences [15], obtained either by direct S measurements or by division of experimental permeability coefficients by experimental diffusion coefficients (S = P/D).

Conformations of polymer chains are the result of molecular mechanical modeling, the procedure of which is implemented in the RDKit (Python) environment. The developed SPCSBP method is performed using freely distributed software, is maximally automated, has the possibility of parallelization on a cluster and has an acceptable calculation time for single polymer. The method also ensures the stability of the obtained results and their reproducibility, and is applicable for specific polymers used in membrane gas separation.

Schematic flowchart of the method is provided in Figure 4 below.

Mean absolute percentage errors (MAPEs) (Equation (6)) and mean percentage errors (MPEs) (Equation (7)) were calculated for all of the prediction methods used in order to assess these methods accuracies and compare them, as follows:

(6) $$MAPE=\frac{100\%}{n}\overset{n}{\underset{i=1}{\sum }}\left|\frac{A_{t}-F_{t}}{A_{t}}\right|,$$

(7) $$MPE=\frac{100\%}{n}\overset{n}{\underset{i=1}{\sum }}\left|\frac{A_{t}-F_{t}}{A_{t}}\right|$$

where *A_t_* is the actual value, *F_t_* is the forecast value and *n* is the number of data points.

## 3. Results and Discussion

### 3.1. Thermal Properties and Free Volume

All the polymers synthesized in the work have high glass transition temperatures (T_g_) (Table 2). The T_g_ for poly(diphenylene phthalide) C4 is not detected and, apparently, it is higher than the decomposition temperature (440 °C). At the same time, according to thermomechanical analysis (TMA) data [67], a softening temperature of 420 °C is observed for this polymer. Poly(diphenylene phthalide) C4, in fact, is a fragment of poly(arylene ether ketone) C3, without the –O–Ph–(C=O)–Ph–O– group; therefore, in a number of synthesized polyheteroarylenes, it is interesting to trace not only the influence of substituents in the main chain, but also the change in its nature. Thus, the traditional replacement of –C(CH_3_)_2_– (C1) with a more voluminous fluorine-containing group, –C(CF_3_)_2_– (C2), leads to an increase in FFV and T_g_ (Table 2). It is obvious that the presence of trifluoromethyl groups in the structure of the polymer C2 should reduce the interchain interactions [68] in the polymer, compared to C1, and, subsequently, reduce the T_g_. However, this effect is inferior to the influence of the chain stiffness, which ultimately causes a slight increase in the glass transition temperature for the polymer C2.

The introduction of a rigid and polar phthalide group (C3) into the main chain of PAEK also leads to an increase in FFV compared to C1, approaching that for C2 (Table 2). The presence of a polar group should increase the interchain interactions in the C3 polymer, hindering molecular mobility, and thereby causing an increase in the glass transition temperature. Therefore, a significant increase in T_g_ for the C3 polymer, compared to the glass transition temperatures for C1 and C2, seems reasonable (Table 2).

The exclusion of the PAEK –O–Ph–(C=O)–Ph–O– group from the main chain is the reason for a significant increase in the chain stiffness; therefore, despite the polarity of the phtalide group, the free volume of the polymer C4 increases significantly. Thereby, the free volume of the polymers increases in the following series: C1 C3 C2 C4.

### 3.2. Experimental Gas Transport Properties

The experimental gas permeability coefficients for the obtained polymer films are presented in Table 3. As can be observed from Table 3, poly(diphenylene phthalide) C4, which can be attributed to medium-permeability polymers, is characterized by the highest P values for all gases. In general, the P values for all gases change in the same manner as the free volume, and naturally increase in the following series: C1 C3 C2 C4. 

Slightly different patterns are observed for the gas diffusion coefficients in the synthesized polymers (Table 4). Poly(diphenylene phthalide) C4 is characterized by the highest values of D, in accordance with the value of fractional free volume. The diffusion coefficients for O_2_, N_2_, CO_2,_ and CH_4_ of C2 are 2.3–3 times less, of C1 are 6–10 times less, and of C3 are 7–12 times less than that of C4. For penetrants with a small molecule size, the differences in the diffusion coefficients are significantly smaller. So, the value of D(H_2_) for C2 is only 1.9 times less than that for C4, and for C1 and C3 it is 3 times less. In general, D for all gases increases in the following series: C3 C1 C2 C4. However, it is worth paying attention to the behavior of the gas diffusion coefficients in PAEK C3. When replacing the –C(CF_3_)_2_– group (C2) with a phtalide moiety (C3), there is a sharp decrease in the diffusion coefficients of permanent gases, by 3–4 times, despite the fact that these polymers have comparable fractional free volumes (Table 4).

It should be noted that the gas permeability and diffusion coefficients for the polymer C3 (Table 3 and Table 4) are consistent with the literature data [69,70].

Generally, as a polymer class, PAEKs show low levels of permeability and diffusion [15]. The bulk phtalide group can be anticipated to increase in terms of its gas permeability and diffusion coefficients. However, there is a significant difference (approximately one order of magnitude) between the P and D of C3 and C4 polymers (Table 3 and Table 4), despite the presence of a phtalide group in both of these structures. This can be attributed to the more-rigid main chain of the C4 polymer, allowing the formation of a kinked structure, which is not the case for the C3 polymer, which contains flexible –O– links. One would expect C4 to show a behavior similar to highly permeable kinked polymers that demonstrate microporosity [12,13,14]. That is not the case though, due to the high dipole moment of the phtalide group that contributes to the high level of interchain interactions for C4.

The behavior of gas permeability and diffusion coefficients has a corresponding effect on the changes in the solubility coefficient S (Table 5) in a series of synthesized polymers. The largest S values for all gases are observed for the polymer C4. However, unlike the data for P and D, the second place, with slightly lower S values for all gases except helium, is occupied by C3. Moreover, the solubility coefficient of CO_2_ for the studied polymers increases in the series C2 C1 C3 C4, while S(N_2_) increases in the series C1 C2 C3 C4. The solubility coefficients of H_2_, O_2,_ and CH_4_ for C1 and C2 are almost the same, so the value of S for the studied polymers increases in the series C1 ≈ C2 C3 C4. For helium, since D is determined with the greatest error, it can only be argued that the solubility coefficient for C4 is greater than that for the others. Basically, this indicates the large size of the free volume element (FVE) in C4 and C3 compared to other polymers, which is probably caused by the presence of a rigid, although polar, phtalide fragment.

**Figure 5:**
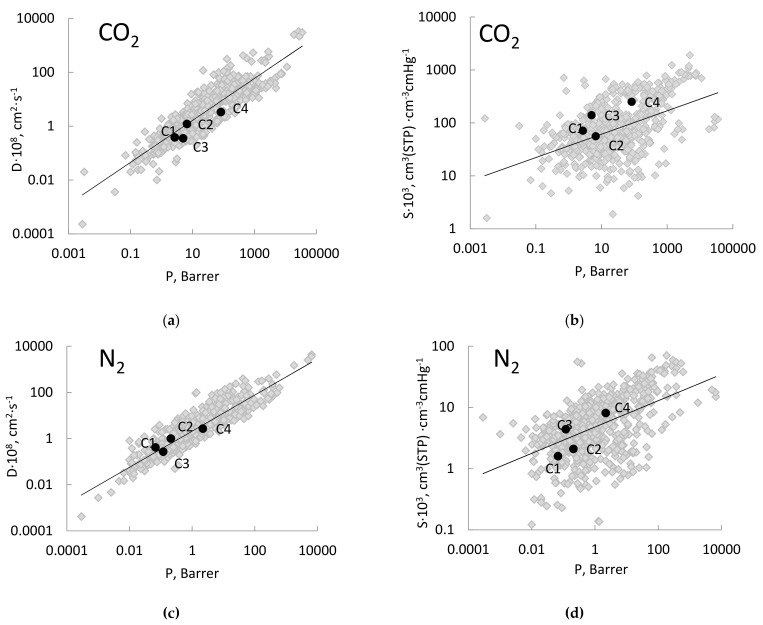
Diagrams of D vs. P and S vs. P for CO_2_ (**a**) and (**b**), respectively) and N_2_ (**c**) and (**d**), respectively). The solid black dots correspond to PAEKs C1–C4, the gray dots correspond to P, D and S of the gases for polymers from TIPS database [15].

A qualitative comparison of the gas transport parameters of the studied PAEKs C1–C4 with the results obtained for other polymers from the TIPS RAS database can be made by plotting them on the diffusion coefficient–permeability coefficient and solubility coefficient–permeability coefficient diagrams [25]. Since the most attractive results for PAEKs were obtained for the CO_2_/N_2_ pair, these diagrams are presented for carbon dioxide (Figure 5a,b) and nitrogen (Figure 5c,d). As can be observed from the presented data (Figure 5a,c), the diffusion coefficients of nitrogen and carbon dioxide naturally increase with the increase in gas permeability. However, while the data points for N_2_ are located near the “middle line” (least-squares fit for overall dataset on the D = f(P) diagram) of the general point cloud, D(CO_2_) for polymers are below the “middle line”, which indicates reduced carbon dioxide diffusion coefficients for the corresponding gas permeability level, especially for the PAEKs C3 and C4. Apparently, this is due to increased interchain interactions in polymers containing a phthalide group.

On similar S vs. P dependencies (Figure 5b,d), the solubility coefficients of N2 for the polymers C3 and C4 are located above the "middle line", while for polymers with a bisphenol fragment (C1 and C2), they are located below it. Thus, for polymers C3 and C4, the influence of an increased free volume is evident (Table 2). It is noteworthy that the solubility coefficients of carbon dioxide for all the studied polymers lie above the "middle line", with the exception of the polymer with the –C(CF_3_)_2_– group (Figure 5b). This behavior seems to be associated not only with an increased free volume in polymers, but also with the presence of specific interactions of the CO_2_ molecule with oxygen-containing phthalide and ketone groups. A similar phenomenon is often recorded when studying sorption and permeability in polymers containing ether groups (e.g., Pebax [71]).

It is worth noting that when replacing the –C (CH_3_)_2_– fragment (C1) with –C (CF_3_)_2_– (C2), despite the increase in the solubility coefficient of N_2_ (Figure 5d), there is a significant decrease in S(CO_2_) (Figure 5b), which may indicate a reduced affinity of the polar CO_2_ molecule to the fluorine-containing group.

**Figure 6:**
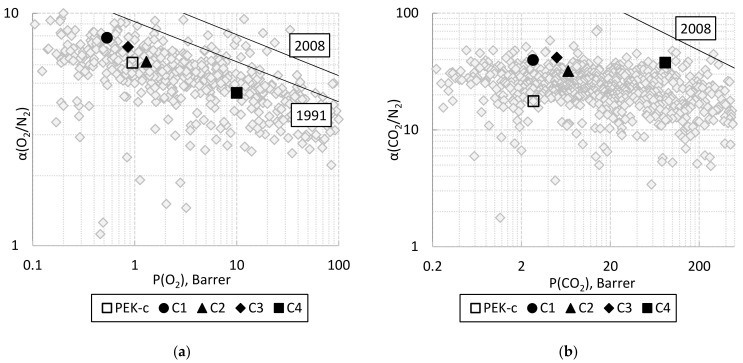
Robeson diagrams for oxygen–nitrogen (**a**) and carbon dioxide–methane (**b**) for the studied polymers. For comparison, the data for poly(arylene ether ketone) PEK-c are given [69] (data from [69] are similar to those from [70]). Upper bounds are provided from [7] (1991) and [8] (2008).

The ideal gas separation selectivities for all the polymers are shown in Table 6; the locations of the studied polymers in the Robeson diagrams for O_2_/N_2_ and CO_2_/N_2_ gas pairs are shown in Figure 6a,b, respectively.

As can be observed from Table 6 and Figure 6a, the most permeable C4 has the lowest selectivity for the oxygen–nitrogen gas pair. The selectivity of polymers increases parallel to the upper bound in the diagram, which corresponds to the trade-off effect. In general, the selectivities of the studied polymers are low, with the exception of the CO_2_/N_2_ pair (Figure 6b). In this diagram, the point for C4 is located near the 2008 upper bound [8] for glassy amorphous polymers. Note that according to the diagrams presented in Figure 5, a high selectivity for the CO_2_/N_2_ pair for the polymers C1, C3 and C4 should be determined by the increased solubility of carbon dioxide.

### 3.3. Predicted Gas Transport Parameters

Table 7 present a comparison of the experimental data for the studied polymers extrapolated to 35 °C by the method from [64], and the data predicted by the chemical structure of the elementary unit by the methods of modified atomic contributions and bond contributions [38] at 35 °C.

As can be observed from Table 7, the experimental and predicted permeability coefficients for C2 and C3, in general, are close, while for C1 and C4, the predicted values are lower than the experimental ones.

It has been previously shown that the BC method is more accurate, as it discriminates the bonds between the main chain and side groups that contribute significantly to polymer rigidity [59]. We can observe the same picture in Table 7, as follows: MAPEs and MPEs are mainly lower for BC, except for the C2 polymer. This may be due to a shared tendency of MAC and BC to overestimate increments for fluorine-containing fragments and, as a result, overestimate P for fluorine-containing polymers [72,73,74].

Though the MAC and BC methods are completely statistical, there is a way to explain some differences in the predictions for C1–C4 polymers via increment values [72]. Let us consider C1/C2 and C3/C4 polymer pairs and the less-accurate MAC method. It can be observed that the permeability coefficient is predicted to be higher for C2 than for C1, which is consistent with the experimental data. Here, one can assume that the –CF_3_ group introduction into the structure as these groups decreases the interchain interactions [68] and leads to the polymer free volume being higher. This is indeed so for the predicted values, as the –CF_3_ side group has a high positive contribution to P, while the –CH_3_ side group has a slight negative contribution to P. It also may be the reason why the error of the C1 prediction is higher; according to the literature [75,76,77,78,79,80,81,82], –CH_3_ introduction into a polymer structure affects gas permeability in an unclear manner, even the position of the –CH_3_ group introduced affects gas permeability in opposite ways [81]. So, there is a high possibility that this ambiguity leads to the –CH_3_ group increment being underestimated, which, in turn, will give a lower level of predicted permeability for polymers where –CH_3_ introduction is increasing the permeability.

It is also worth mentioning that the model used splits the polymer in a manner where –CH_3_ is a single side-group atom (with hydrogen neighboring atoms), while –CF_3_ is a side-group C atom (with non-hydrogen neighboring atoms) and three side-group F atoms. Returning to Figure 3, this is the moment that should be considered carefully for optimization of the splitting process used. The software now does not discriminate between the –F atom being a substituting atom at the fluorinated phenyl-group or –CF_3_ group, and does not discriminate between side-group C atoms in the tert-butyl group or –CF_3_ group. However, it is important to perform analysis showing if it is appropriate or not.

As for the C3–C4 pair, first, it is worth mentioning that data from [69,70] were used in the training dataset, so it is only expected that the predictions for this polymer will be more accurate. Both models (MAC and BC) predict C4 permeability to be higher than that of C3, which is in accordance with the measured permeability coefficients. However, the ratio P(C4)/P(C3) for the actual values is significantly higher than that for the predicted ones (Table 7). The difference between the C3 and C4 repeating units is in two additional phenyl-rings in C3, and also in the –O– and C=O links in the main chain of the latter (Table 2). Phenyl rings do not lead to a high difference, because their higher number in C3 is compensated by normalization on molecular weight. So, –O– and C=O fragments seem to contribute to the predicted permeability difference. As –O– and C=O (combined C= and =O atoms) have negative contributions to gas permeability, C3 permeability being lower than that of C4 seems logical. However, the ratio P(C4)/P(C3) difference can only be explained by the overestimation of negative –O– and =O absolute values (C= fragment has a positive value, so it is out of the question), and underestimation of other fragments positive increments. These should be slightly modified, so that they were compensated in the same way as they are now in the C3 structure, while making C4 (where no –O– and non-phtalide =O are presented) permeability higher. It leads us to the need of closer increments investigation, in order to make MAC and BC prediction methods more accurate and universal. Table 8 presents the experimental gas diffusion coefficients D·10^8^ (cm^2^·s^−1^) in synthesized polymers at 35 °C, and predicted data for the same polymers.

**Figure 7:**
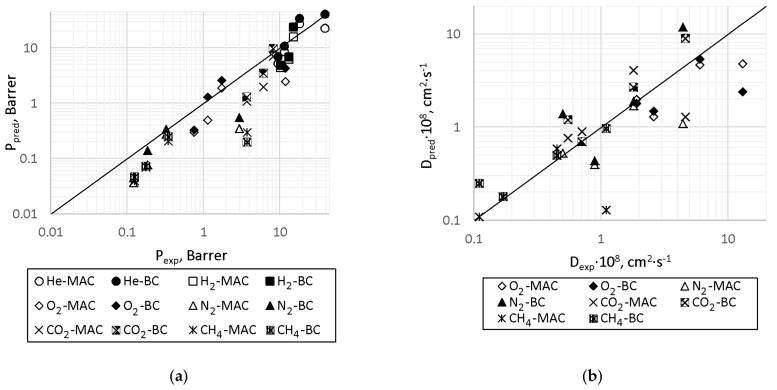
Comparison diagram of predicted and experimental values of permeability coefficient (**a**) and diffusion coefficient (**b**).

As can be observed from Table 8, the predicted diffusion coefficients for all the PAEKs (C1–C3) are close to the experimental ones, and for C4, there is a large spread between the two methods; BC, in general, predicts larger D, while MAC predicts the smaller ones. In this case, the BC method does not show the expected accuracy, as there are more different increments for this method than for MAC, and it leads to the equation system being less overdetermined than is needed for the method to be precise. Overall, the database [15] does not contain as many diffusion coefficient values as permeability coefficient values, so the prediction accuracy drops [38]. Figure 7 shows the comparison of experimental and predicted data in the diagrams P_pred_ = f(P_exp_) (Figure 7a) and D_pred_ = f(D_exp_) (Figure 7b).

As can be observed from Figure 7, generally, the scatter of the experimental and predicted data for C1–C3 fits into the half order of magnitude value, which, essentially, corresponds to an average accuracy of predictions for MAC for separate classes of polymers [29,32,35], and exceeds the accuracy of the predictions as MAC and BC for the entire dataset [38]. The predicted values for C4 are out of the range of the half order of magnitude, but remain within the range of one order of magnitude deviation, inherent of the accuracy of the predictions of both MAC and BC over the entire dataset [38]. Apparently, this behavior for C4 is caused by the abnormal rigidity of the polymer and the lack of representatives of such a class of polymers in the whole dataset, which naturally affects the accuracy of predictions by the group contribution method.

The solubility coefficients, predicted using the SPCSBP method, are presented in Table 9. These values were compared to the solubility coefficients calculated by the MAC and BC methods (Table 9), as P_pred_/D_pred_, where P_pred_ and D_pred_ are the predicted permeability and diffusion coefficients, respectively, obtained via the MAC or BC methods. The experimental solubility coefficients, obtained as P/D (both parameters extrapolated to 35 °C [64]), are also present in Table 9. It should be noticed that the extrapolation of P and D to 35 °C was performed by the method from [64], using weak correlations (R^2^ = 0.25–0.66), which might lead to a high error in the P (35 °C) and D (35 °C) calculation. Therefore, the S (35 °C) calculation error may also be high. However, to our knowledge, the method from [64] is the only method to extrapolate experimental values of P and D to 35 °C when temperature dependences are unavailable.

It is worth noting that the MAPEs and MPEs presented in Table 7, Table 8 and Table 9 were calculated for each of the four polymers investigated. MAPEs were also calculated for individual regressions for different gases using all the validation set of polymers. They were the following: He—100%, H_2_—56%, O_2_—53%, N_2_—59%, CO_2_—67%, CH_4_—77%. It can be observed that though the overall MAPEs are satisfactory, some disturbingly large MAPEs were obtained for some of the individual polymers (MAPE for C1 is 175% and for C2 is as great as 362%).

**Figure 8:**
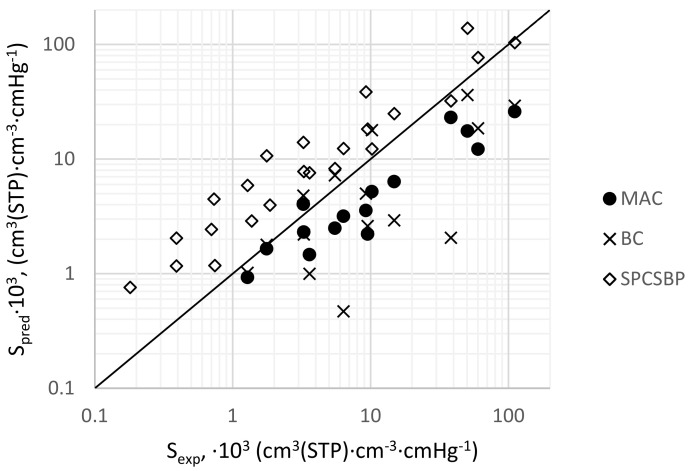
Comparison diagram of predicted and experimental values of solubility coefficient.

The comparison diagram S_pred_ = f(S_exp_) is presented in Figure 8.

As is demonstrated in Figure 8, the MAC/BC and SPCSBP methods predictions are within one order of magnitude (except two points obtained by the BC method) compared to the experimental values. This level of prediction accuracy is not quite satisfactory for the solubility coefficient; however, some interesting observations can be made for further development of the methods. It is worth mentioning that MAC/BC and SPCSBP methods have shown opposite deviations; mostly negatively in the case of MAC/BC and mostly positively in the case of SPCSBP. Using the MPE calculation results, one can clearly see that the mostly underestimated (positive MPE numbers) values of P_pred_ combined with the overestimated (negative MPE numbers) values of D_pred_ lead to the expected noticeable S underestimation. 

The SPCSBP method has more physical sense, as it includes longer-chain fragments modeling, and is based on the theory connecting polymer and gas surface areas with gas solubility in polymers [83]. However, if one compares the prediction errors for the polymers under discussion, it is obvious that SPCSBP gives good solubility coefficient predictions for C3 and C4, while there are significant errors observed for C1 and C2. The main difference between C3–C4 and C1–C2 polymers is in the presence of a phtalide group in the former pair, and the presence of a –C(CH_3_)_2_ or –C(CF_3_)_2_ group in the latter. We have calculated MAPE for poly(arylene ether ketone)s with a phtalide group and without it present in the training dataset used. They were 93% and 14% for –C(CH_3_)_2_ or –C(CF_3_)_2-_containing and phtalide-containing poly(arylene ether ketone)s, respectively. It seems that the error of the method is also persistent for –C(CH_3_)_2_ or –C(CF_3_)_2_-containing poly(arylene ether ketone)s, because MAPE for such polymers is six times higher than for the others. A possible explanation of the phenomenon might be associated with the circumstances that (i) the indices used in the SPCSBP method do not describe the peculiarities of the –C(CH_3_)_2_ or –C(CF_3_)_2_ groups in poly(arylene ether ketone)s structures, and their effect on gas solubility; (ii) experimental data for poly(arylene ether ketone)s with such fragments is not sufficient for regression training. All of these issues demand further investigation. This will include the addition of new fragment indices into the regression, application of the clustering method from [65] with regression building for separate clusters, and also comparison of different oligomer conformations obtained by the SPCSBP method and molecular modeling method from [84,85,86,87].

## 4. Conclusions

The poly(arylene ether ketone)s and polyarylene with propylidene and phtalide moieties, with high glass transition temperatures and fractional free volumes, were synthesized. The introduction of hexafluoropropylidene (C2) and phtalide (C3) groups was shown to increase the glass transition temperature, gas permeability, and fractional free volume, when compared to the C1 polymer, due to the increase in the rigidity of the polymer chain and interchain interactions. It was demonstrated that a bulky phtalide group acts differently when introduced into a rigid or flexible main chain, and can lead to the formation of a kinked structure in the former case. However, increase of permeability and diffusion coefficients for such kinked structure is limited compared to well-known kinked polymers (PIM-like, Tröger base polymers, iptycene-based polymers, etc.), due to an increase in the interchain interactions caused by the high dipole moment of the phtalide group and compaction of the structure. Group contribution methods (MAC and BC) demonstrated (i) the same qualitative trends for the predicted gas permeabilities and diffusivities as the ones observed for the experimental data, and (ii) reasonable agreement of the predicted parameters with the experimental ones for all the polymers under investigation, except polydiphelenephtalide (C4). The latter is attributed to the overestimation of negative increments of –O– and =O fragments and the underestimation of positive increments of other fragments, present in the C4 polymer. The gas solubility coefficients obtained by the SPCSBP method have sufficient forecasting power for phtalide-containing polymers, while the opposite is true for propylidene-based PAEKs. This fact demands additional investigation of the influence of –C(CH_3_)_2_ and –C(CF_3_)_2_ bridges in the structure of polymers of different classes on geometrical indices in the framework of the SPCSBP approach.

## Figures and Tables

**Table 1:** Distribution of polymers throughout classes.

Class	Number of Polymers
Polyimides	394
Polyacetylenes	107
Polyesters	88
Copolymers	81
Polyethers	72
Polynorbornenes (metathesis and additive)	70
Polysulfones	52
Polyamidoimides	40
Polyamides	37
Other nitrogen-containing polymers	31
Polystyrenes	22
Polycarbonates	19
Vinyl polymers	15
Polyacrylates	15
Other carbon-chain polymers	10
Other hetero-chain polymers	8
Polyphosphazenes	4

**Table 2:** Physicochemical properties of PAEKs.

Polymer Designation	Structure	T_g_, °C	ρ, g/cm^3^	FFV, %
C1	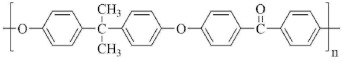	155	1.195	10.4
C2	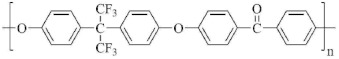	163	1.377	11.9
C3	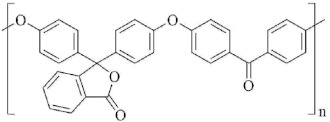	229 218 [69] 249 [70]	1.259 1.249 [69] -	11.6 12.3 [69] -
C4	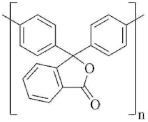	440	1.215	13.3

**Table 3:** Experimental gas permeability coefficients P (Barrer ^1^) for synthesized polymers at (21 ± 2 °C).

Polymer	He	H_2_	O_2_	N_2_	CO_2_	CH_4_
C1	6.8	7.0	0.53	0.068	2.7	0.065
C2	14	11	1.3	0.21	6.7	0.22
C3	8.9	9.7	0.86	0.12	5.0	0.11
C4	32	52	10	2.2	83	2.8

^1^ Barrer = 10^−10^ cm^3^(STP)·cm·cm^−2^·s^−1^·cmHg^−1^.

**Table 4:** Experimental gas diffusion coefficients D·10^8^ (cm^2^·s^−1^) in synthesized polymers at (21 ± 2 °C).

Polymer	He	H_2_	O_2_	N_2_	CO_2_	CH_4_
C1	290	76	1.5	0.41	0.38	0.067
C2	290	120	3.8	1.0	1.2	0.23
C3	230	74	1.2	0.27	0.35	0.056
C4	360	230	8.9	2.7	3.3	0.68

**Table 5:** Experimental gas solubility coefficients S·10^3^ (cm^3^(STP)·cm^−3^·cmHg^−1^) for synthesized polymers at (21 ± 2 °C), calculated as P/D.

Polymer	He	H_2_	O_2_	N_2_	CO_2_	CH_4_
C1	0.23	0.92	3.6	1.6	71	9.7
C2	0.48	0.94	3.4	2.1	56	9.6
C3	0.39	1.3	7.2	4.4	140	20
C4	0.89	2.3	11	8.1	250	41

**Table 6:** Different gas pairs ideal selectivities (α) of permeability for synthesized polymers at (21 ± 2 °C).

Polymer	α						
	O_2_/N_2_	CO_2_/N_2_	CO_2_/CH_4_	He/N_2_	H_2_/CH_4_	He/CH_4_	H_2_/N_2_
C1	7.9	40	42	100	110	100	100
C2	6.2	32	30	67	50	63	52
C3	7.2	42	45	74	88	81	81
C4	4.5	38	30	15	19	11	24

**Table 7:** Experimental gas permeability coefficients P (Barrer) for synthesized polymers at 35 °C and predicted data for the same polymers.

Polymer	Type of Data	He	H_2_	O_2_	N_2_	CO_2_	CH_4_	*MAPE*^1^, %	*MPE*^2^, %
C1	Experimental	9.33	10.1	0.753	0.120	3.67	0.124	-	-
	MAC	5.3	4.5	0.30	0.037	1.1	0.040	61	61
	BC	7.1	4.9	0.33	0.045	1.3	0.047	53	53
C2	Experimental	17.9	15.0	1.72	0.320	8.16	0.345	-	-
	MAC	28	16	1.9	0.28	7.2	0.21	23	−1.7
	BC	35	24	2.6	0.34	9.8	0.25	43	−34
C3	Experimental	11.4	13.1	1.13	0.183	6.08	0.175	-	-
	MAC	8.7	6.3	0.50	0.078	2.0	0.070	53	53
	BC	11	6.9	1.3	0.14	3.5	0.073	32	27
C4	Experimental	38.4	64.5	11.8	2.93	89.2	3.71	-	-
	MAC	23	22	2.5	0.35	8.6	0.30	76	76
	BC	41	46	4.3	0.56	10	0.20	61	58

^1^*MAPE* = mean absolute percentage error. ^2^ *MPE* = mean percentage error.

**Table 8:** Experimental gas diffusion coefficients D·10^8^ (cm^2^·s^−1^) in synthesized polymers at 35 °C and predicted data for the same polymers.

Polymer	Type of Data	O_2_	N_2_	CO_2_	CH_4_	*MAPE*, %	*MPE*, %
C1	Experimental	2.6	0.89	0.71	0.17	-	-
	MAC	1.3	0.4	0.9	0.18	34	12
	BC	1.5	0.44	0.7	0.18	25	15
C2	Experimental	6.0	1.8	1.8	0.45	-	-
	MAC	4.7	1.7	4.1	0.59	45	−21
	BC	5.4	1.9	2.7	0.50	18	−9
C3	Experimental	1.9	0.5	0.55	0.11	-	-
	MAC	2	0.53	0.77	0.11	13	−8
	BC	1.8	1.4	1.2	0.25	107	−69
C4	Experimental	13	4.4	4.6	1.1	-	-
	MAC	4.8	1.1	1.3	0.13	75	50
	BC	2.4	12	9.0	0.97	91	−28

**Table 9:** Experimental gas solubility coefficients S·10^3^ (cm^3^(STP)·cm^−3^·cmHg^−1^) in synthesized polymers at 35 °C and predicted data for the same polymers.

Polymer	Type of Data	He	H_2_	O_2_	N_2_	CO_2_	CH_4_	MAPE ^1^, %	*MPE*^2^, %
C1	Experimental	0.25	1.0	2.9	1.3	52	7.3	-	
	MAC	-	-	2.3	0.93	12	2.2	50	33
	BC	-	-	2.2	1.0	19	2.6	44	30
	SPCSBP	0.76	2.4	7.8	5.9	77	18	175	−175
C2	Experimental	0.49	1.0	2.9	1.8	44	7.7	-	
	MAC	-	-	4.0	1.7	18	3.6	40	13
	BC	-	-	4.8	1.8	36	5.0	30	−3
	SPCSBP	2.1	4.5	14	11	140	39	362	−362
C3	Experimental	0.40	1.4	5.9	3.7	110	15	-	
	MAC	-	-	2.5	1.5	26	6.4	63	42
	BC	-	-	7.2	1.0	29	2.9	63	34
	SPCSBP	1.2	2.9	8.3	7.6	100	25	86	−84
C4	Experimental	0.89	2.3	9.1	6.6	190	33	-	
	MAC	-	-	5.2	3.2	66	23	47	65
	BC	-	-	18	0.47	11	2.1	94	64
	SPCSBP	1.2	4.0	12	12	140	32	42	−32

## Data Availability

Not applicable.

## Supplementary Materials

The following are available online at https://www.mdpi.com/article/10.3390/membranes11090677/s1. Table S1. Chemical shifts and signal multiplicity in ^1^H NMR spectra for samples C1–C4. Table S2. Chemical shifts and signal multiplicity in ^13^C NMR spectrum for samples C1–C4.
