# Supplementary material for: Structure-Property Relationship on the Example of Gas Separation Characteristics of Poly(Arylene Ether Ketone)s and Poly(Diphenylene Phtalide)
Source: Membranes (Basel). 2021 Aug 31;11(9):677. doi: 10.3390/membranes11090677 (PMC8465416; doi:10.3390/membranes11090677)
Supplement: Supplementary file 1 [file membranes-11-00677-s001.zip › membranes-1335385-supplementary.pdf]

membranes

Supplementary Material

Structure-Property Relationship on the Example of Gas Separation Characteristics of Poly(Arylene Ether Ketone)s and Poly(Diphenylene Phtalide)

Alexandre Alentiev [1,*], Sergey Chirkov [1], Roman Nikiforov [1], Mikhail Buzin [2], Oleg Miloserdov [3], Victoria Ryzhikh [1], Nikolay Belov [1], Vera Shaposhnikova [2], and Sergey Salazkin [2]

[1] A.V. Topchiev Institute of Petrochemical Synthesis, Russian Academy of Sciences (TIPS RAS), 119991 Moscow, Russia
[2] A.N.Nesmeyanov Institute of Organoelement Compounds, Russian Academy of Sciences (INEOS RAS), 119334 Moscow, Russia
[3] V.A. Trapeznikov Institute of Control Sciences, Russian Academy of Sciences (ICS RAS), 117997 Moscow, Russia
* Correspondence: alentiev@ips.ac.ru; Tel.: +7 916 201 68 55

Table S1. Chemical shifts and signal multiplicity in ^{1}H NMR spectra for samples C1-C4.

δ_H, ppm			
C1 [1]<br>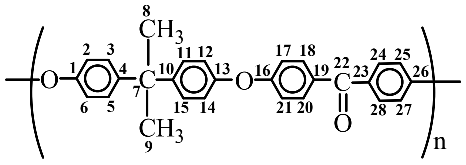	C2<br>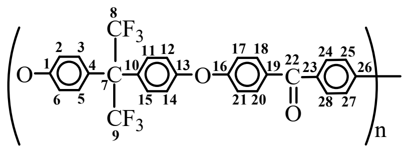	C3 [1]<br>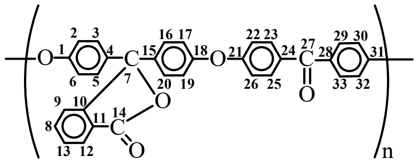	C4 [2]<br>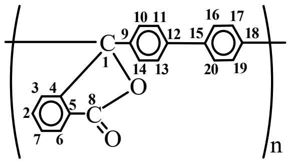
1.72 s, 6 H, H(8), H(9),<br>7.00 d, 4 H, H (2), H(6), H(12), H(14), $^3J_{H-H}$=8.3 Hz;<br>7.03 d, 4 H, H (17), H(21), H(25), H(27), $^3J_{H-H}$=8.3 Hz;<br>7.28 d, 4 H, H (3), H(5), H(11), H(15), $^3J_{H-H}$=8.3 Hz;<br>7.79 d, 4 H, H (18), H(20), H(24), H(28), $^3J_{H-H}$=8.3 Hz;	7.11 d, 4 H, H (2), H(6), H(12), H(14), $^3J_{H-H}$=8.0 Hz;<br>7.13 d, 4 H, H (17), H(21), H(25), H(27), $^3J_{H-H}$=8.0 Hz;<br>7.46 d, 4 H, H (3), H(5), H(11), H(15), $^3J_{H-H}$=8.0 Hz;<br>7.88 d, 4 H, H (18), H(20), H(24), H(28), $^3J_{H-H}$=8.0 Hz;	7.02 d, 4 H, H(2), H(6), H(17), H(19), $^3J_{H-H}$=8.8 Hz;<br>7.04 d, 4 H, H (22), H(26), H(30), H(32), $^3J_{H-H}$=8.8 Hz;<br>7.37 d, 4 H, H (3), H(5), H(16), H(20), $^3J_{H-H}$=8.8 Hz;<br>7.58 t, 1 H, H (13), $^3J_{H-H}$=7.6 Hz;<br>7.60 d, 1 H, H (9), $^3J_{H-H}$=7.6 Hz;<br>7.73 t, 1 H, H (8), $^3J_{H-H}$=7.6 Hz;<br>7.77 d, 4 H, H (23), H(25), H(29), H(33), $^3J_{H-H}$=8.8 Hz;<br>7.95 d, 1 H, H (12), $^3J_{H-H}$=7.6 Hz;	7.43 d, 4 H, H(10), H(14), H(17), H(19),, $^3J_{H-H}$=8.3 Hz;<br>7.52 d, , 4 H, H(11), H(13), H(16), H(20), $^3J_{H-H}$=8.3 Hz;<br>7.58 dd, 1 H, H (7), $^3J_{H-H}$=7.6; 7.7 Hz;<br>7.63 d, 1 H, H (3), $^3J_{H-H}$=7.7 Hz;<br>7.73 dd, 1 H, H (2), $^3J_{H-H}$=7.6; 7.7 Hz;<br>7.96 d, 1 H, H (6), $^3J_{H-H}$=7.7 Hz;

Table S2. Chemical shifts and signal multiplicity in ^{13}C NMR spectrum for samples C1-C4.

Atom*				
	C1 [1]	C2	C3 [1]	C4 [2]
C(1)	161.04	160.10	155.91	91.28
C(2)	118.37	118.90	117.55	134.41
C(3)	131.65	132.36	128.76	126.21
C(4)	146.36	128.80	132.43	151.73
C(5)	131.65	132.36	128.76	125.52
C(6)	118.37	118.90	117.55	124.17
C(7)	41.98	63.87	90.72	129.62
C(8)	30.68	122.70	134.24	196.62
C(9)	30.68	122.70	123.82	140.11
C(10)	146.36	128.80	151.54	127.67
C(11)	131.65	132.36	125.22	127.26
C(12)	118.37	118.90	129.46	140.52
C(13)	161.04	160.10	126.00	127.26
C(14)	118.37	118.90	169.24	127.67
C(15)	131.65	132.36	132.43	140.52
C(16)	153.07	156.65	128.76	127.26
C(17)	114.68	118.32	117.55	127.67
C(18)	127.39	132.00	155.91	140.11
C(19)	131.77	133.04	117.55	127.67
C(20)	127.39	132.00	128.76	127.26
C(21)	114.68	118.32	160.33	
C(22)	193.69	194.12	119.40	
C(23)	131.77	133.04	132.04	
C(24)	127.39	132.00	136.39	
C(25)	114.68	118.32	132.04	
C(26)	153.07	156.65	119.40	
C(27)	114.68	118.32	193.84	

C(28)	127.39	132.00	136.39	
C(29)			132.04	
C(30)			119.40	
C(31)			160.33	
C(32)			119.40	
C(33)			132.04	

* See Table S1 for the designation of atoms.

[1] Shaposhnikova, V. V.; Salazkin, S. N.; Donetskii, K. I.; Gorshkov, G. V.; Sharapov, D. S.; Mamedova, I. A.; Petrovskii, P. V.; Askadskii, A. A.; Bychko, K. A.; Kazantseva, V. V.; Krasnov, A. P.; Afonicheva, O. V. Synthesis and Properties of Cardo Copoly(Arylene Ether Ketones). *Polymer Science - Series A* **2002**, *44* (6), 563–569.
[2] Fatykhov, A. A.; Sedova, E. A.; Egorov, A. E.; Salazkin, S. N.; Kraikin, V. A. NMR Study of Phthalide-Type Poly(Phenylene)s. Symmetry and Additivity: NMR Study of Phthalide-Type Poly(Phenylene)s. Symmetry and Additivity. *Magn. Reson. Chem.* **2017**, *55* (10), 958–966. https://doi.org/10.1002/mrc.4613.
